# HLA-DO and Its Role in MHC Class II Antigen Presentation

**DOI:** 10.3389/fimmu.2013.00260

**Published:** 2013-08-29

**Authors:** Yuri O. Poluektov, AeRyon Kim, Scheherazade Sadegh-Nasseri

**Affiliations:** ^1^Graduate Program in Immunology, Johns Hopkins University, Baltimore, MD, USA; ^2^Department of Pathology, Johns Hopkins School of Medicine, Baltimore, MD, USA

**Keywords:** HLA-DO, HLA-DR antigens, MHC class II antigen processing, models for HLA-DO function, HLA-DM

## Abstract

Helper T cells are stimulated to fight infections or diseases upon recognition of peptides from antigens that are processed and presented by the proteins of Major Histocompatibility Complex (MHC) Class II molecules. Degradation of a full protein into small peptide fragments is a lengthy process consisting of many steps and chaperones. Malfunctions during any step of antigen processing could lead to the development of self-reactive T cells or defective immune response to pathogens. Although much has been accomplished regarding how antigens are processed and presented to T cells, many questions still remain unanswered, preventing the design of therapeutics for direct intervention with antigen processing. Here, we review published work on the discovery and function of a MHC class II molecular chaperone, HLA-DO, in human, and its mouse analog H2-O, herein called DO. While DO was originally discovered decades ago, elucidating its function has proven challenging. DO was discovered in association with another chaperone HLA-DM (DM) but unlike DM, its distribution is more tissue specific, and its function more subtle.

## Introduction

Major Histocompatibility Complex (MHC) Class II molecules are heterodimeric glycoproteins consisting of one α-chain of approximately 34 kDa and one β-chain of approximately 28 kDa (1). In general, polymorphic classical MHC class II molecules bind and present peptide antigens. Non-classical MHC II molecules are generally non-polymorphic and serve as chaperones and accessory proteins that assist with folding, transport, antigenic peptide loading, and editing (2). An inherent difficulty in studying the MHC II locus arose from the large genomic distances between the protein coding regions of some alpha and beta chain pairs. When the beta chain of the DO molecule was discovered it was originally thought to be another member of the classical MHC II family and not an accessory molecule, and hence was designated as Aβ2, an alternative beta chain of the existing MHC II I-A molecule (3). While the classical β-chains were 63–69% homologous to each other, Aβ2 chain was found to have 49–56% homology to the A and E β-chains and to the human DP, DQ, and DR β-chains. This made the Aβ2 chain the most divergent member of the β-chain family. Another difference between the Aβ2 gene and classical MHC II genes was that it showed very little polymorphism. This led to the hypothesis that Aβ2 may have a function distinct from the classical Aβ and Eβ genes.

It took many years before the protein product of the Aβ2 gene (at the time referred to as H2-Oβ) was finally confirmed by a study that made a rabbit antiserum against the predicted cytoplasmic tail. Two-dimensional electrophoreses of immunoprecipitated H2-Oβ indicated the presence of an α chain. H2-O was found to be expressed only in tissue samples of B cell dependent areas and a few sparse locations in the thymic medulla, leading to some speculation about its role in the immune system (4). The human form of DO was discovered in the same way. This study in addition to confirming the existence of DO in humans reported that DO interacted stably with DM and could be co-precipitated as a complex (5). It was found that the transport of DO chains out of the ER depended upon its oligomerization with DM. The necessity of DM for transport was supported by the poor expression of H2-O in DM knockout mice, as in the absence of DM no DO could be detected outside the ER. It seemed strange that DO would require a stable interaction with DM for the sole purpose of being shuttled to the endosomal compartment (6, 7). Recently it was reported that the assembly of DM/DO complex depended upon a single residue in the alpha chain, a buried Proline (alpha11) on the floor of the putative peptide-binding groove (8). Authors demonstrated that mutating this residue abolished all effects of DM on ER egress and intracellular trafficking. The existence of DO in complex with DM became a new paradigm in research on DO, leading to the postulation that DO must be a regulator of DM functions.

## Tissue Distribution of HLA-DO/H2-O

Another key indicator of the biological significance of DO is its differential tissue distribution. Unlike all other MHC II molecules, which are fully expressed in professional Antigen Presenting Cells (APCs), DO was originally detected only in B cells and the thymic medulla (4). Expression of DO in B cells has been shown to vary throughout cellular development and activation (9, 10). Immature mouse bone marrow B cells show expression of conventional MHC II molecules as well as DM, but not DO. Only after migration to the spleen do B cells start to show the detectable levels of DO which are maintained in all transitional B cell subsets and even in mature cells. However, this expression level is significantly down-regulated upon entry into germinal centers (GCs).

Further studies using human cells found that HLA-DR (DR) and DO were expressed in thymic medullary cells (11, 12). Specifically, the epithelial cells, which ring the Hassall’s corpuscles (HC) structures, expressed high levels of DO. HCs are unique structures found within the thymic medulla, of varying size and morphology, consisting of “swirls” of keratinized epithelium (13). The HC bodies are the only sites within the human thymic medulla where dying thymocytes are detected outside of the thymic cortex. Not much is known about the true purpose of HCs in the human immune system, although some studies have linked them to the generation of regulatory T cells and autoimmune disease (14).

More recently, few studies reported some expression of DO in certain subsets of DCs (10, 15). Most notable is the BDCA-3^+^ subset of human blood plasmacytoid DCs, which nearly uniformly expresses DO (16). DO was also found to be expressed in subpopulations of BDCA1^+^ CD11c+ DCs and tonsillar interdigitating DCs. The expression of DO in Langerhans cells (LCs) was found to vary greatly among donors, which diminished upon maturation. In another study, CD8α^+^ murine splenic DCs expressed more H2-O than CD8α^−^ DC (15). It is apparent that the up- or down-regulation of DO is largely regulated by the immune system in various tissues, an indication that DO contributes in a unique way to antigen processing and presentation.

## HLA-DO/H2-O Functions Observed *In vivo*

DM knockout mice produced detectable phenotypes of altered antigen presentation (17–21). Most notable phenotype in the first DM^−/−^ mice was the predominant occupancy of their MHC II (I-Ab) with CLIP peptides. H-2M^−/−^ mice had a reduced number of CD4+ T cells, which reacted strongly against wild-type cells in mixed lymphocyte reaction. With DO, the story turned out to be much more complicated. It was observed that human T cell lines when transfected with genes for DR1, DM, and DO presented CLIP at higher levels than when DO was absent, suggesting that DO inhibited the function of DM (22). On the contrary, B cells from DO knockout mice expressed CLIP at similar levels as their wild-type counterparts (23, 24). Throughout countless experiments, the effects of DO on antigen presentation appeared highly controversial, if detectable at all, as the *in vivo* results did not match the findings in transfected cells lines (23–25). One notable study interrogated the ability of B cells in H2-O knockout and wild-type mice to enter the GCs (26). Given that the expression of DO in B cells was up-regulated during maturation but down-regulated upon the entry into GCs, the study reasoned that DO may be affecting the ability of B cells to enter the GCs. If DO had a measurable effect on the presentation of Class II antigens on the cell surface, it would also affect the ability of B cells to receive CD4^+^ T cell help and hence enter the GCs. To test this hypothesis a 1:1 mixture of H2-O^−/−^ and wild-type B cells specific for the 4-hydroxy-3-nitrophenyl acetyl ligand (NP) were adoptively transferred to B6 recipient mice. Mice were then immunized with NP-linked chicken gamma globulin (CGG), and then the abundance of H2-O^−/−^ and wild-type B cells in the GCs were measured 20 days later. Draghi et al. (26) found that H2-O^−/−^ cells outnumbered wild-type B cells by a ratio of 3 to 1. The study went to great lengths to confirm that these results were due to enhanced ability of H2-O^−/−^ cells to present CGG. However, when NP-linked ovalbumin (OVA) was used as an antigen, the effect was reversed. This time wild-type B cells expressing DO outnumbered the DO knockout cells in the GCs. This study demonstrated that the effect of DO on antigen presentation could vary depending on the antigen. When detectable, the effect of DO on the presentation of two antigens that were tested was not “*all-or-none*,” but rather incremental; a large portion of the B cell population was favored over the other.

In an attempt aimed at discerning the effects of DO, Yi et al. overexpressed human HLA-DO genes in CD11c^+^ DCs of non-obese diabetic (NOD) mice (27). The results were astonishing, as HLA-DO transgenic mice (NOD.DO) did not develop diabetes even after 50 weeks. However, when NOD.DO T cells were transferred into NOD.*SCID* hosts lacking T and B cells, mice developed diabetes. More importantly, NOD.DO mice developed diabetes upon receiving T cells from diabetic NOD donors, indicating that DO overexpression in DCs did not prevent diabetogenic T cells from forming but prevented their pathogenic effects. A more recent study reported that H2-O^−/−^ mice could spontaneously develop high titers of antinuclear antibodies (ANAs) indicative of a mouse model of autoimmune systemic lupus erythematosus (28). The mice did not, however, develop an autoimmune pathology associated with lupus, and H2-O^−/−^ mice showed a reduced capacity to present exogenous antigens to the helper T cells, adding to the contradictory nature of DO.

## HLA-DM/HLA-DO Complex Crystal Structure

To avoid the complexities associated with the *in vivo* experiments, a need for direct biochemical and structural studies became evident. A breakthrough was made when a 3-D crystal structure of the DM/DO complex was solved (29). The study showed that binding of DM to DO reduced the ability of DM to enhance binding of a variant of HA-306-318 peptide of influenza of Texas77 to DR1, and to dissociate CLIP. Authors demonstrated that upon binding to DO, the conformation of DM does not change significantly, as compared to unbound DM (30, 31). These findings were further enforced with the recent crystal structure of the DM-DR complex (32, 33). The structure of DO in complex with DM superimposed well with the structure of DR1 (34). Altogether, the structural evidence suggests that DO may mimic the MHC class II molecules in binding to DM, possibly to compete with MHC class II interacting with DM. It is important to note that even though the interaction between DM and DO is stable, the interaction between DM and DR is transient and was only stabilized under highly stringent conditions, which included generation of peptide-receptive DR molecules (35–37). While the interface of DM interacting with DR might be the same as that of DM interacting with DO, the magnitude and the functional specificity of this interaction are completely different. Mutational analysis that mapped residues important to the interaction of DM with DR1 showed that the amino acid residues known to disturb the DM/DR interaction (38) mapped almost entirely to the DM/DO interface (39). However, although DM mutants, DM βHis141 and βSer142, inhibited the ability of DO to suppress DM, they had no effect on the ability of DM to facilitate peptide binding to DR, presumably because of differences in the nature of the two interactions (33).

## Mechanisms of HLA-DO Function

While many different models have been put forward to explain the mechanism of DO function, the most dominant one is that of DO being an inhibitor of DM. With the release of the DM/DO crystal structure, this model has gained even more support. The inhibition of DM by DO was observed in some of the earliest biochemical assays performed with DM/DO complexes copurified from cells (22, 40), as well as recombinant soluble DO within pH ranges of 5.5–6, but was absent at the pH of 4.5–5.0 (23). Authors proposed that DO was necessary to limit the pH interval in which DM is fully active. In contrast, other studies did not confirm a pH dependent effect on the inhibitory function of DO (39, 41).

Alternative models were developed to explain DO function. One such model came from Kropshofer et al. who reported that DM/DO complexes purified from the human spleen had a positive effect on the loading of HA peptide onto DR4, DR1, and DR3 molecules as compared to purified DM alone (42). More importantly, the study showed that the enhancement of HA peptide binding occurred even when soluble recombinant DO was used instead of the cell purified molecules. Although this effect of DO was exactly the opposite of those observed previously, it was also pH dependent with DO performing best in the same pH range as described before. But the best insights into the function of DO came from peptide elution experiments from DR4, where addition of DO changed the repertoire of the eluted peptides as compared to DM only (42). In the presence of DM, four out of eight peptides were predominantly loaded onto the DR4 molecules, but when both DM and DO were present, a different set of four out of eight peptides was detected. These findings suggested that just like DM, DO can up- or down-modulate presentation of certain peptides.

Another attempt toward understanding the role of DO in peptide selection was recently published (43). In this study authors found differences in how DO affected loading of different peptides onto DR1. A type II collagen-derived peptide and a low-affinity HA peptide variant were inhibited in their binding to DR1, whereas the bindings of the immunodominant peptides from two different strains of influenza were enhanced by the addition of DO (44). Authors found that DO only affected the association of peptides to DR1 and had no effect on their dissociation. Moreover, the differential effects of DO on peptide association directly correlated with DR1 peptide complex sensitivity to DM-mediated dissociation. Peptides that formed complexes with DR1 and were sensitive to DM-mediated dissociation were inhibited by DO, whereas those peptide complexes that were resistant to DM-mediated dissociation were enhanced by DO. Importantly, the effect of DO was dominant over the effects of DM. Adding to the complexity was the observation that the effect of DO on peptide binding occurred even in the absence of DM. Since DO only affected the association of peptide binding, and that DO is always in complex with DM in the antigen processing compartments, authors theorized that DO might only affect certain open conformations of DR molecules generated by DM. A signature of MHC class II molecules is their uncommon tendency to adopt different conformations based on binding to peptides of different sequences and/or conformational states associated with transient interactions with accessory molecules. It follows that for MHC II molecular interactions with DM and the following peptide selections all depend on the recognition of different conformations of the molecules (35–37).

To test the model that DO might only affect certain open conformations of DR molecules, Poluektov et al. examined the effects of DO on peptide binding to a mutant DR1 molecule that is always in an open peptide-receptive conformation (45–47) independent of DM. The mutant molecule although resistant to recognition by DM, was inhibited from binding to a DM-sensitive HA peptide variant in the presence of DO. Considering that the mutant DR1 molecule is unable to adopt a closed conformation and is fixed in its receptive state, it is likely that DO forces the DR1 molecule into yet another conformation that preferentially binds DM-resistant peptides, which can generate a closed compact folded MHC II. As such the effects of DO would be to enhance the binding of DM-resistant peptides while diminishing binding of the DM-sensitive peptides.

**Figure 1:**
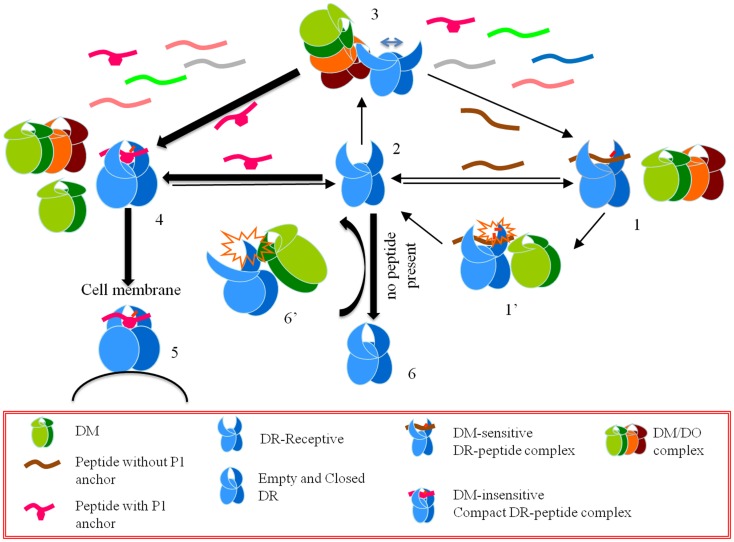
**A model for the effects of HLA-DO on antigen presentation**. Starting from a CLIP-bound DM-sensitive conformation (*conformation 1*), DR interacts with DM (*conformation 1’*), and a peptide-receptive open conformation is generated (*conformation 2*). An open conformation can also be induced by DM interacting with empty DR (*conformation 6’*). DO or DM/DO complexes interact with peptide-receptive DR molecules and stabilize an overly receptive conformation (*conformation 3*). In the pool of available peptides those that form DM-sensitive complexes with DR do not get a chance to stabilize in the groove. On the contrary, those peptides that form DM-resistant complexes undergo conformational changes and form DR-compact dimers (*conformation 4*), which are shuttled to the cell membrane (*conformation 5*). If DR-receptive (conformation 2) does not find a peptide to bind it converts to a closed conformation (*conformation 6*).

Considering the data above, Poluektov et al. proposed a model for the mechanism of DO in the context of DM and DR (Figure 1). Authors proposed that DO works together with DM to edit the peptide repertoire presented by MHC II. In this model DM is necessary to generate a peptide-receptive conformation in DR, which is then acted upon by DO. DM thereby provides the first round of peptide editing and DO selects from the peptides favored by DM by promoting the binding of DM-resistant peptides while inhibiting the binding of DM-sensitive ones. To verify the model, authors showed that DO forms a stable complex with a peptide-receptive DR molecule using surface plasmon resonance. In addition, co-expressed recombinant DM/DO had the same effects on peptide binding as DO alone, even when the DM/DO complexes were used in conjunction with free DM. Based on the activity of the DM/DO complex reported by Poluektov et al. the active site of DO is likely located outside the interface between DM and DO, and hence DM/DO has the same functionality as that of DO alone.

## Conclusion

While the true mechanism of DO still remains largely unknown, based on the data available to us we can speculate on its role in the immune system. We know that DO is a highly evolutionarily conserved molecule (48–50), but unlike the classical MHC genes, DO is not polymorphic. This indicates that DO is in fact an important gene that provides some evolutionary benefits to its host. It has even been reported that a point mutation in the human HLA-DOα gene was linked to a susceptibility to rheumatoid arthritis (RA) (51), suggesting that the effects of DO are far reaching.

While the model that describes DO solely as an inhibitor of DM by preventing its major role in the selection of immunodominant epitopes (44, 52) fits some of the observed effects of DO (53), it does not provide an adequate explanation for why DO has been evolutionary so conserved nor does it explain the variable expression of DO among different subsets of professional APCs, and at different stages of cellular differentiation (8, 54). A more likely explanation is that DO has some critical role in fine tuning, or improving the antigenic peptide repertoire selection in collaboration with DM: the expression of DO in B cells could easily be explained by the need to limit the number of long-lived memory T cells developed against different antigens (55–57), and in the thymus, DO contributes to the presentation of a repertoire that is most contrived yet effective permitting deletion of all possible self-reactive T cells (58). DO is potentially a new lever that could help to control the antigenic repertoires generated by the immune system.

## Conflict of Interest Statement

The authors declare that the research was conducted in the absence of any commercial or financial relationships that could be construed as a potential conflict of interest.
